# An increase in cesarean section rate during the first wave of COVID-19 pandemic in Iran

**DOI:** 10.1186/s12889-023-15907-1

**Published:** 2023-05-24

**Authors:** Maryam Gharacheh, Mohammad Ebrahimi Kalan, Narjes Khalili, Fahimeh Ranjbar

**Affiliations:** 1 Nursing and Midwifery Care Research Center, Iran University of Medical Sciences, Rashid Yasemi St., Valiasr Ave., P.O. Box 19395-4798, Tehran, Iran; 2 School of Health Professions, Eastern Virginia Medical School, Norfolk, VA USA; 3 Lineberger Comprehensive Cancer Center, University of North Carolina at Chapel Hill, Chapel Hill, NC USA; 4 Department of Community and Family Medicine, School of Medicine, Preventive Medicine and Public Health Research Center, Psychosocial Health Research Institute, Iran University of Medical Sciences, Tehran, Iran

**Keywords:** COVID-19, Cesarean section, Maternal health

## Abstract

**Background:**

The COVID-19 pandemic and its impact on healthcare services is likely to affect birth outcomes including the delivery mode. However, recent evidence has been conflicting in this regard. The study aimed to assess changes to C-section rate during the COVID-19 pandemic in Iran.

**Methods:**

This is a retrospective analysis of electronic medical records of women delivered in the maternity department of hospitals in all provinces of Iran before the COVID-19 pandemic (February-August 30, 2019) and during the pandemic (February-August 30, 2020). Data were collected through the Iranian Maternal and Neonatal Network (IMAN), a country-wide electronic health record database management system for maternal and neonatal information. A total of 1,208,671 medical records were analyzed using the SPSS software version 22. The differences in C-section rates according to the studied variables were tested using the χ2 test. A logistic regression analysis was conducted to determine the factors associated with C-section.

**Results:**

A significant rise was observed in the rates of C-section during the pandemic compared to the pre-pandemic (52.9% vs 50.8%; *p* = .001). The rates for preeclampsia (3.0% vs 1.3%), gestational diabetes (6.1% vs 3.0%), preterm birth (11.6% vs 6.9%), IUGR (1.2% vs 0.4%), LBW (11.2% vs 7.8%), and low Apgar score at first minute (4.2% vs 3.2%) were higher in women who delivered by C-section compared to those with normal delivery (*P* = .001).

**Conclusions:**

The overall C-section rate during the first wave of COVID-19 pandemic was significantly higher than the pre-pandemic period. C-section was associated with adverse maternal and neonatal outcomes. Thus, preventing the overuse of C-section especially during pandemic becomes an urgent need for maternal and neonatal health in Iran.

## Synopsis

### Study question

How has the first wave of COVID-19 pandemic affected the C-section rate in Iran?

### What’s already known

The COVID-19 pandemic and its impact on healthcare services may have adverse impacts on maternal and perinatal health including the delivery mode.

### What this study adds

The overall C-section rate during the first wave of COVID-19 pandemic was significantly higher compared to the pre-pandemic period.

## Background

The Coronavirus disease 2019 (COVID-19) pandemic has affected the health of pregnant women globally [1], and its full impact on maternal outcomes remained unknown [2]. Earlier evidence has been conflicting concerning the effect of COVID-19 pandemic on obstetric interventions such as cesarean section (CS) [3]. Despite the lack of evidence for vertical transmission and no clinical evidence for this mode of delivery [4], the status of COVID-19 alone became a common indication for C-section early in the pandemic both in the infected and non-infected pregnant women [5–8]. It seems that concerns about intrapartum vertical transmission of COVID-19 infection and fears of contracting COVID-19 at hospitals may have led to changes in decision making on the mode of delivery in early pandemic [9]. The nationwide lockdowns limited providing prenatal care and counseling to pregnant women and considerably raised anxiety among pregnant women and healthcare professionals. The unpredictability of labor may also affect the decision making on the mode of delivery for both pregnant women and obstetricians [1, 8]. The increase in C-section rates may also reflect the efforts of obstetricians to provide the best services to their patients in the face of constantly changing guidelines regarding the safest delivery method for the mother, baby, and provider [8, 10].

Although, the increased risk for C-section has been reported during the pandemic, this risk appears to be limited to women with severe or critical disease and being infected in third-trimester [11]. Adverse outcomes such as ICU admission or patient death may occur but the clinical course of COVID-19 is not severe in most women, and the infection does not significantly affect pregnancy [4]. In fact, in most cases the disease does not threaten the mother. Therefore, COVID-19 is not considered an indication for an elective C-section [4, 12], and the mode of delivery should be individualized based on the severity of the disease and obstetrical indications [13]. However, C-sections without medical indications have regularly increased globally [14], and further research on the changing C-section trend during the pandemic is also needed [15]. Iran has had one of the highest rates of C-section (47.9%) in the world before the COVID-19 pandemic [14], and this dramatic rise in cesarean birth is rooted in the medicalization of birth [16]. Iran is a country located in the Middle East and is the 18th largest country in the world, with a population of 83.70 million in 2020 [17]. The primary healthcare services, such as antenatal care and immunization, are free of charge, and most of the secondary and tertiary health services in Iran are provided by the public sector [18]. As of 28 March, there were 423.03 cases of COVID-19 per million population and 30.07 deaths per million confirmed in Iran. At this time, the case fatality rate was estimated about 7.5%, which was twice as high as the global average [17]. Since the beginning of the pandemic, the Iranian government started undertaking strict interventions in order to curb the pandemic including the cancelation of public events, closure of schools, universities, and shopping centers, as well as outposts for identifying COVID-19 at city entrances [19].

To the best of our knowledge, there is little evidence regarding the rate of C-section during the first wave of COVID-19 in Iran. Since improving the maternal outcomes is a global priority, especially during a pandemic, the present nationwide study was conducted to assess how the first wave of COVID-19 pandemic has affected the C-section rate in all provinces of Iran.

## Methods

### Study design

The study is part of a comprehensive national study on reproductive health outcomes during the COVID-19 pandemic which was conducted in 2020 in Iran. We conducted a retrospective analysis of electronic medical records of women delivered in the maternity department of hospitals in all provinces of Iran before the COVID-19 pandemic (February 30 to August 30, 2019) and during the pandemic (February 30 to August 30, 2020), covering the first wave of the pandemic. To collect data, we used a data retrieval form on socio-demographic and fertility-related characteristics and the reproductive health outcomes through the Iranian Maternal and Neonatal Network (IMAN) that is a country-wide electronic health record database management system for maternal and neonatal information. In this study, outcome measures were C-section rates, and perinatal outcomes of C-section. Data were exported to Excel® and statistical analyses were performed using the SPSS software version 22.

Comparisons between pre and intra-COVID-19 pandemic involved six months of the same period of the year to take into account the possible influence of seasonal differences. The differences in C-section rates according to the studied variables were tested using the χ2 test. A logistic regression analysis was conducted to determine the factors associated with C-section. A *P* value less than 0.05 were considered statistically significant.

### Ethical approval

Ethical approval for the research project was obtained from the ethics committee of the National Institute for Medical Research Development (Code: IR.NIMAD.REC.1399.076). All methods were performed in accordance to the Declaration of Helsinki. Given that we only used the women’s medical records and were not in touch with any women, it was not possible for us to obtain informed consent from the subjects. However, to ensure confidentiality, no participant was recorded by name, and the results were only reported in groups.

## Results

A total of 1,208,671 medical records were analyzed in the study with 624,521 (51.7%) before the COVID-19 pandemic and 584,150 (48.3%) during the pandemic. Table 1 indicates the C-section delivery rates during the pandemic and pre-pandemic periods by province. Gilan (75%), and Tehran (72%) had the highest rates of C-section delivery, and Sistan and Baluchestan had the lowest rate of C-Sect. (26%) during the pandemic.

**Table 1:** C-section rates during the pandemic and pre-pandemic periods by province

Province	Pandemic period N (%)	Pre-pandemic period N (%)	*P*-value
Alborz	9075 (57.7%)	8963 (53.8%)	.001
Ardabil	5886 (61.3%)	6049 (57.8%)	.001
East Azerbaijan	17,249 (65.7%)	17,687 (62.3%)	.001
West Azerbaijan	13,393 (50.7%)	12,856 (47.2%)	.001
Bushehr	3822 (47.9%)	3993 (46.9%)	.183
Chahar Mahaal and Bakhtiari	3571 (47.1%)	3424 (44.3%)	.001
Fars	18,962 (58.3%)	19,761 (56.5%)	.001
Gilan	7969 (75.0%)	7971 (69.5%)	.001
Golestan	8933 (56.1%)	8683 (51.5%)	.001
Hamadan	6226 (49.7%)	6381 (47.2%)	.001
Hormozgan	6969 (42.2%)	7153 (41.1%)	.050
Ilam	1977 (54.4%)	2097 (53.2%)	.286
Isfahan	18,728 (57.3%)	19,360 (54.6%)	.001
Kerman	12,378 (49.9%)	12,319 (46.5%)	.001
Kermanshah	6345 (47.9%)	6511 (46.8%)	.074
North Khorasan	2421 (34.9%)	2752 (36.4%)	.058
Razavi Khorasan	24,567 (42.2%)	25,688 (41.1%)	.001
South Khorasan	3100 (39.7%)	3212 (39.7%)	.043
Khuzestan	20,993 (47.5%)	20,993 (45.7%)	.001
Kohgiluyeh Boyer-Ahmad	2498 (46.8%)	2471 (43.3%)	.001
Kurdistan	4171 (35.8%)	4119 (34.0%)	.003
Lorestan	6658 (49.8%)	6975 (49.9%)	.952
Markazi	3397 (44.3%)	3487 (42.8%)	.059
Mazandaran	10,404 (67.6%)	11,820 (68.9%)	.012
Qazvin	4611 (55.9%)	4699 (53.4%)	.001
Qom	5438 (55.6%)	5438 (54.3%)	.051
Semnan	2467 (57.1%)	2534 (54.8%)	.033
Sistan and Baluchestan	9104 (26.0%)	8318 (24.2%)	.001
Tehran	58,458 (72.2%)	59,898 (68.5%)	.001
Yazd	5525 (50.0%)	5960 (48.7%)	.041
Zanjan	3841 (48.0%)	3702 (43.0%)	.001

**Fig. 1:**
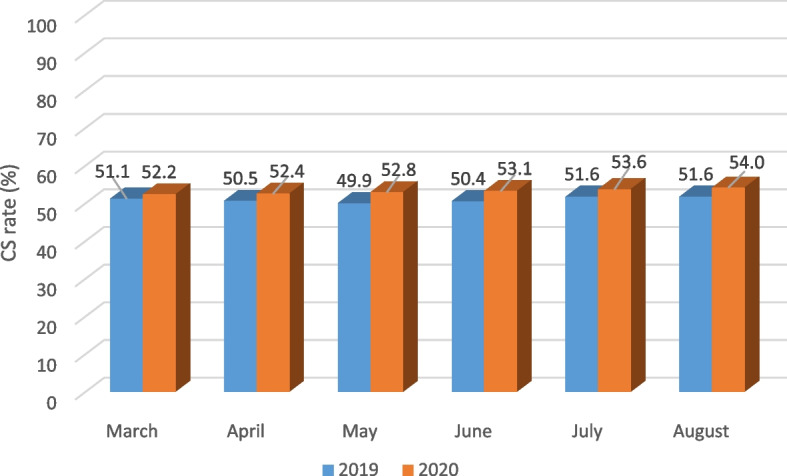
Monthly rate of C-section delivery during the two periods of study

The characteristics of women with C-section compared with those with normal delivery during the pandemic and pre-pandemic periods are presented in Table 2. The mean age of mothers was 29.38 ± 6.13 years. Most mothers had the education level of secondary school. The mean ± SD gestational age was 38.26 ± 2.10 weeks. Neonates had a mean ± SD birth weight of 3920 ± 0.450 g. About 98.3% and 1.7% of pregnancies were singleton and multiple pregnancy, respectively. Out of 584,150 deliveries during the pandemic, 309,136 (52.9%) were delivered by C-section. In contrast, during the pre-pandemic, there were 624,521 deliveries, 317,333 of which (50.8%) were delivered by C-section. A significant rise was observed in the rates of C-section during the pandemic compared to the pre-pandemic (*p* = 0.001). Figure 1 displays an increasing trend in the C-section rates during the pandemic period. Regarding the place of delivery, 75.4% of C-section deliveries (vs 24.6% NVD) took place in private hospitals and 44.9% (vs 55.1% NVD) in public hospitals (*p* = 0.001).

**Table 2:** Characteristics of women with C-section delivery compared with those with normal delivery during the pandemic and pre-pandemic periods

Characteristics		Pandemic period *N* = 584,150					Pre-pandemic period *N* = 624,521				
		Normal delivery *N* = 307,188		CS delivery *N* = 317,333		*p*-value	Normal delivery *N* = 275,014		CS delivery *N* = 309,136		*p*-value
		N	%	N	%		N	%	N	%	
Maternal age (year)	≤ 30	177,252	54.4	148,506	45.6	.001	202,314	56.4	156,417	43.6	.001
	> 30	97,762	37.8	160,630	62.2		104,874	39.5	160,916	60.5	
Gestational age (week)	37–42	255,338	48.3	272,827	51.7	.001	284,990	50.4	280,020	49.6	.001
	< 37	18,997	34.6	35,947	65.4		21,469	36.8	36,933	63.2	
	> 42	679	65.2	362	34.8		729	65.7	380	34.3	
Parity	1	94,641	44.1	120,116	55.9	.001	101,414	44.5	126,357	55.5	.001
	≥ 2	79,543	53.6	68,782	46.4		84,846	54.8	69,969	45.2	
Abortion	0	221,764	48.5	235,184	51.5	.001	249,989	50.8	242,139	49.2	.001
	≥ 1	53,250	41.9	73,952	58.1		57,199	43.2	75,194	56.8	
Living children	0	104,127	45.6	124,284	54.4	.001	125,553	49.9	126,289	50.1	.001
	1	94,344	44.1	119,729	55.9		100,821	44.6	125,129	55.4	
	≥ 2	76,543	54.0	65,123	46.0		80,814	55.1	65,915	44.9	
Mother’s education	Illiterate	16,309	48.4	17,393	51.6	.001	23,869	69.7	10,377	30.3	.001
	Primary education	76,562	46.9	86,523	53.1		58,547	62.2	35,519	37.8	
	Secondary education	104,062	46.6	119,314	53.4		168,104	50.9	162,395	49.1	
	Academic	78,081	47.6	85,906	52.4		56,668	34.2	109,042	65.8	
Birth weight (gr)	< 2500	17,990	36.6	31,215	63.4	.001	20,613	39.2	31,926	60.8	.001
	2500–4000	249,416	48.3	267,153	51.7		278,784	50.4	274,795	49.6	
	> 4000	7608	41.4	10,768	58.6		7785	42.3	10,612	57.7

The logistic regression analysis indicated that maternal age > 30 years (*P* = 0.001), the mother’s education level of secondary school (*P* = 0.001), abortion (*P* = 0.001), gestational age < 37 weeks (*P* = 0.001), and birthweight < 2500 gr (*P* = 0.001) and > 4000 gr (*P* = 0.001) were associated with higher chances of C-section delivery during the pandemic and pre-pandemic periods (Table 3). Table 4 indicates that repeated C-sections and fetal distress were the two commonest causes of C-section deliveries.

**Table 3:** Multiple logistic regression analysis of factors associated with C-section delivery during the pandemic and pre-pandemic periods

Variable		Pandemic period				Pre-pandemic period			
		AOR	95% CI for OR		*P* value	AOR	95% CI for OR		*P* value
			Lower	Upper			Lower	Upper	
Maternal age (year)		2.46	2.43	2.49	.001	1.09	1.090	1.092	.001
Gestational age (week)	37–42	1				1			
	< 37	1.56	1.53	1.60	.001	1.57	1.53	1.60	.001
	> 42	.60	.53	.69	.001	.74	.64	.84	.001
Parity		.81	.80	.83	.001	.82	.80	.83	.001
Abortion		1.22	1.21	1.24	.001	1.13	1.12	1.13	.001
Living child		.88	.87	.90	.001	.94	.93	.96	.001
Birth weight (gr)	2500–4000	1				1			
	< 2500	1.18	1.15	1.21	.001	1.20	1.17	1.23	.001
	> 4000	1.38	1.34	1.43	.001	1.42	1.37	1.46	.001
Mother’s education	Illiterate	1				1			
	Primary education	1.04	1.02	1.07	.001	1.22	1.18	1.25	.001
	Secondary education	1.05	1.03	1.08	.001	1.98	1.93	2.03	.001
	Academic	1.02	1.00	1.05	.050	3.04	2.96	3.12	.001

^*^P < 0.05 *AOR* Adjusted odds ratio, *CI* Confidence interval

**Table 4:** The indications for the C-section during the pandemic and pre-pandemic periods

Indications	Pandemic period		Pre-pandemic period	
	N	%	N	%
Previous C-section	159,661	51.6	169,727	53.5
Fetal distress	41,124	13.3	40,954	12.9
Mal-presentation	17,870	5.8	19,317	6.1
Placenta/cord problems	5640	1.8	5924	1.9
Maternal request	19,824	6.4	17,384	5.5
Dystocia	12,147	3.9	13,689	4.3
Multiple pregnancy	11,698	3.8	17,412	5.5
Other	41,173	13.4	32,926	10.3

Pregnancy outcomes of women with C-section delivery compared with those with normal delivery during the pandemic and pre-pandemic periods are presented in Table 5. The rates for preeclampsia, gestational diabetes, preterm birth, IUGR, LBW, and low Apgar score at first minute were higher in women who delivered by C-section compared to those with normal delivery (*P* = 0.001). The multiple logistic regression analysis showed that preterm birth (*P* = 0.001), gestational diabetes (*P* = 0.001), preeclampsia (*P* = 0.001), IUGR (*P* = 0.001), LBW (*P* = 0.001), and 1-min Apgar score < 7 (*P* = 0.001) were associated with higher chances for C-section delivery.

**Table 5:** Pregnancy outcomes of women with C-section delivery compared with those with normal delivery during the pandemic and pre-pandemic periods

Characteristics	Pandemic period					Pre-pandemic period				
	Normal delivery *N* = 307,188	C-section delivery *N* = 317,333	AOR	95% CI for OR	*p*-value	Normal delivery *N* = 275,014	C-section delivery *N* = 309,136	AOR	95% CI for OR	*p*-value
	N (%)	N (%)				N (%)	N (%)			
Preterm birth	18,997 (6.9)	35,947 (11.6)	1.67	1.63–1.71	.001	21,469 (7.0)	36,933 (11.6)	1.65	1.64–1.68	.001
Gestational diabetes	8376 (3.0)	18,951 (6.1)	1.96	1.91–2.01	.001	8679 (2.8)	18,276 (5.8)	1.98	1.93–2.01	.001
Preeclampsia	3606 (1.3)	9340 (3.0)	1.90	1.82–1.97	.001	3737 (1.2)	9072 (2.9)	1.93	1.85–2.00	.001
IUGR	1136 (0.4)	3565 (1.2)	2.18	2.04–2.34	.001	1488 (0.5)	3955 (1.2)	2.05	1.92–2.18	.001
LBW	21,334 (7.8)	34,597 (11.2)	1.15	1.13–1.18	.001	24,237 (7.9)	35,344 (11.1)	1.11	1.09–1.13	.001
1-min Apgar score < 7	9263 (3.4)	13,070 (4.2)	1.64	1.57–1.71	.001	9976 (3.2)	13,980 (4.4)	1.84	1.76–1.91	.001
5-min Apgar score < 7	6288 (2.3)	5273 (1.7)	.26	.25–28	.001	6653 (2.2)	5286 (1.7)	.25	.24–27	.001

^*^P < 0.05 *AOR* Adjusted odds ratio, *CI* Confidence interval

## Discussion

The findings of this study revealed an overall significant rise in the C-section rate from 50.8% in the pre-pandemic period to 52.9% during the first wave of COVID-19 pandemic, and the rates were increasing over time. Similarly, a study by Bhatia et al. from England showed a rise in the overall C-section rate during the pandemic (28.3% to 29.7%) [17]. Iran has one of the highest rates of C-section in the world. The prevalence of C-section has been reported to be 48% [18]. Notwithstanding the Iranian Ministry of Health has imposed policies to reduce the rates of C-section including implementing mother-friendly hospitals, providing protocols for labor pain relief, and holding workshops for midwives and obstetricians, these measures have not been highly successful due to the medicalization of childbirth in Iran [16]. The increase in the C-section rate observed in our study may be explained by the fact that during the COVID-19 pandemic, C-section delivery has been preferred to ensure a controllable delivery process, and avoid emergency respiratory problems [19]. C-section as a quicker procedure reduces the time women spend waiting for giving birth, and therefore decreases the exposure time to the hospital environment during the pandemic [7]. It is also postulated that due to the pandemic, more women might have chosen the certainty of a C-section date rather than awaiting spontaneous labor. The strong demand for C-section delivery rather than normal delivery might be influenced by women’s negative emotions from the panic of contracting COVID-19 [8]. In general among pregnant population, fears of contracting COVID-19 at health centers, and difficulties in transportation during the lockdown has led to a reluctance in timely referrals to emergency care departments. These facts can contribute to a delay in the management of pregnancy complications, and in turn to a rise in preterm deliveries, with an increased risk of C-section in this context [1]. Previous studies suggest that reducing access to maternity care may increase the rates of C-section [6, 20]. According to Verhoeven et al., women more preferred to give birth at home during the COVID-19 pandemic [21]. Lack of home birth facilities in Iran might have caused more women to choose C-section during this period. Furthermore, inadequate access to normal delivery services for women who had a previous C-section may have contributed to a rise in the C-section rate during the pandemic. Although COVID-19 should not be considered as an indication for C-section [22], studies have reported an increase in the C-section rate globally [4, 8, 23]. According to the recent evidence, placental infection during COVID-19 results in placental vascular disease, IUGR, and higher risks of perinatal death [24–26]. Fear of these eventual adverse events could add to higher rates of iatrogenic preterm delivery using C-section [1]. However, Eleje et al. showed an overall decline in C-section rates during the COVID-19 pandemic compared to the pre-pandemic period. The authors discussed that obstetricians might have hesitated in performing C-section due to continued monitoring with C-section only performed for absolute indications [15]. An analysis of women delivering at New York City hospitals found no changes in C-section rates during the first wave of COVID-19 compared to the pre-pandemic period [12]. Overall, changes to the delivery mode during the pandemic have been reported with mixed findings [27].

As of 28 March 2020, Iran reported 35,408 confirmed cases of COVID-19 and 2517 total associated deaths, and ranked 7th in the list of confirmed cases globally. Therefore, the COVID-19 lockdown was imposed across the country in March and April 2020 [28]. We observed an increasing trend in the C-section rates particularly following the lockdown. These findings are likely to be partially attributed to the restriction of movement, and delays in presentation for childbirth, and changes in prioritizing non-urgent health services during the lockdown. Subsequently after the lockdown, there may have been changes in obstetricians’ behaviors towards a reduced threshold for interventions to expedite deliveries and to avoid emergency situations [23].

Although repeated C-sections and fetal distress were the two top indications for C-section in our study, C-section on maternal request increased during the pandemic. Also, 13.4% of C-sections had no indications. These might be normal pregnancies which ended in C-sections without medical indications and on maternal request. Li et al. also reported an increase in C-section on maternal request during the COVID-19 pandemic [24]. Globally, common indications for C-section are cephalopelvic disproportion (CPD) followed by a previous history of C-section [29]. A repeated C-section is reported as a major risk factor for delivering by C-section in subsequent pregnancies in countries with high rates of C-section that can be modified with appropriate policies [30]. There were variations in the C-section rates between geographical regions of Iran with higher rates observed in metropolitan cities, which possibly reflect different practices and capacities. In our study, more than three-fourths of C-section deliveries took place in private hospitals. COVID-19 restrictions may have led women from a high socioeconomic status to seek care in private facilities due to fear of exposure to COVID-19 in overcrowded and short-staffed public hospitals as well as worries about poor facilities in public sector [31]. Similar to our findings, evidence suggests requests for C-section are observed more commonly in the private sector, and public health facilities are more likely to serve low-middle and low socioeconomic status populations [30, 32].

Iranian women with C-Section differed from those with normal delivery in some variables. They were more likely to be older, primiparous, with lower gestational age, and have newborns with LBW or macrosomia. Higher maternal age [6, 18] and primiparity [33, 34] as factors influencing the C-section rate have been reported in previous research. There is a general consensus that the reported rise in C-section rates is often due to the presence of comorbidities either underlying or induced by pregnancy, such as diabetes, and hypertension [33]. The high rate of high-risk pregnancies, which can result in premature delivery, contributes to a rise in the rates of preterm and LBW babies, with an increased risk of C-section deliveries [35]. Additionally, C-section delivery as a risk associated with macrosomia has been suggested in the literature [36]. During the pandemic, women tend to gain weight due to the lack of enough physical activities and eating more food, which may lead to the failure of weight management [6], and in turn an increased risk of C-section. Further research is required to explore the risk factors affecting maternal health management during pandemic and to provide more specific recommendations [37].

The adverse effects of C-section delivery on maternal and neonatal outcomes have been well documented [8]. We observed higher chances of C-section significantly associated with preterm birth, gestational diabetes, preeclampsia, IUGR, LBW, and low Apgar score. This could be explained by the fact that most pregnant women with maternal complications and medical conditions undergo C-section delivery. Also, a major factor to consider when making the decision whether to perform C-section is the fetal birth weight [38]. A considerable number of complicated pregnancies which necessitates premature termination of pregnancies, such as preeclampsia, preterm premature rupture of membranes, and preterm labor, will have a large number of low birth weight delivery. Thus, there will be higher rates of neonatal complications including low Apgar score [39]. These findings are supported by prior studies [20, 30, 40]. There is evidence that pregnant women experiencing COVID-19 may have higher rates of perinatal and maternal morbidities including preterm birth, preeclampsia, and C-section delivery [23]. On the other hand, during the pandemic, there has been reduced access to routine in-person prenatal care, less monitoring of potential complications, and an increased avoidance of care due to fears of COVID-19 [27]. Further research is needed to understand the underlying mechanisms for these findings.

The main strength of this study can be its large size with including almost all births in urban health centers over two periods of six months, covering the first wave of the COVID-19 pandemic, allowing us to generalize the results to the rest of Iran. However, using registered data for research can be seen as a limitation. We were not able to use the Robson’s classification because we analyzed only the data registered on the IMAN from February 30 to August 30, 2019 and from February 30 to August 30, 2020, and data on some variables required for Robson’s classification (such as presentation) were not available within this time period. Our results were only for the first wave, and further research is needed to target the impacts of the following waves of the pandemic on the rates of C-section. Moreover, due to the nature of the data, the adverse pregnancy outcomes cannot be considered causal since the outcomes may be related to the medical reasons for the C-section [15, 30].

## Conclusions

The C-section rate during the first wave of COVID-19 pandemic was significantly higher than the pre-pandemic period. Results from our study provide evidence that the COVID-19 pandemic has substantially affected C-section practices in hospitals, and challenged obstetric settings in their tendency towards C-section. Our findings will be of importance when looking at healthcare programs aimed to cut high rates of C-sections which are medically unexplained, particularly during pandemics. Considering the increasing trend of C-section rates in Iran, it seems that new policies to reduce C- sections should consider de-medicalization of birth by strengthening the home birth system.

C-section was associated with adverse maternal and neonatal outcomes. Thus, preventing the overuse of C-section especially during pandemic becomes an urgent need for maternal and neonatal health in Iran. Providing individualized support for pregnant women on the most appropriate mode of delivery can reduce the rate of unnecessary C-sections during pandemic. Favoring normal delivery in COVID-19 infected women is paramount, since it reduces the risk of clinical deterioration, and neonatal morbidities related to iatrogenic preterm births [1]. Using midwifery led care in the management of low-risk pregnancies is cost-effective [41], and can be helpful in reducing the rate of C-sections. Since the repeated C-section was the most common indication for C-section, improving the rate of VBAC (vaginal birth after C-section) should be taken into consideration in future policies. Hence, regular monitoring of C-section indications during pandemic, and careful assessment of pregnant women with a repeated C-section to determine the possibility of normal delivery are needed to ensure the appropriate use of the C-section.

## Data Availability

The data that support the findings of this study are available from the research deputy of Iran University of Medical Sciences but restrictions apply to the availability of these data, which were used under license for the current study, and so are not publicly available. Data are however available from the corresponding author upon reasonable request and with the permission of research deputy of Iran University of Medical Sciences.
